# Automated extraction of genes associated with antibiotic resistance from the biomedical literature

**DOI:** 10.1093/database/baab077

**Published:** 2022-01-20

**Authors:** Andre Brincat, Markus Hofmann

**Affiliations:** Department of Informatics, TU Dublin, Blanchardstown Campus, Dublin D15 YV78, Ireland; Department of Informatics, TU Dublin, Blanchardstown Campus, Dublin D15 YV78, Ireland

## Abstract

The detection of bacterial antibiotic resistance phenotypes is important when carrying out clinical decisions for patient treatment. Conventional phenotypic testing involves culturing bacteria which requires a significant amount of time and work. Whole-genome sequencing is emerging as a fast alternative to resistance prediction, by considering the presence/absence of certain genes. A lot of research has focused on determining which bacterial genes cause antibiotic resistance and efforts are being made to consolidate these facts in knowledge bases (KBs). KBs are usually manually curated by domain experts to be of the highest quality. However, this limits the pace at which new facts are added. Automated relation extraction of gene-antibiotic resistance relations from the biomedical literature is one solution that can simplify the curation process. This paper reports on the development of a text mining pipeline that takes in English biomedical abstracts and outputs genes that are predicted to cause resistance to antibiotics. To test the generalisability of this pipeline it was then applied to predict genes associated with *Helicobacter pylori* antibiotic resistance, that are not present in common antibiotic resistance KBs or publications studying *H. pylori.* These genes would be candidates for further lab-based antibiotic research and inclusion in these KBs. For relation extraction, state-of-the-art deep learning models were used. These models were trained on a newly developed silver corpus which was generated by distant supervision of abstracts using the facts obtained from KBs. The top performing model was superior to a co-occurrence model, achieving a recall of 95%, a precision of 60% and F1-score of 74% on a manually annotated holdout dataset. To our knowledge, this project was the first attempt at developing a complete text mining pipeline that incorporates deep learning models to extract gene-antibiotic resistance relations from the literature. Additional related data can be found at https://github.com/AndreBrincat/Gene-Antibiotic-Resistance-Relation-Extraction

## Introduction

The increasing prevalence of antibiotic-resistant bacteria is one of the greatest challenges humanity is currently facing (1). With the discovery of one of the first antibiotics—penicillin, antibiotics became a huge breakthrough that prevented millions of deaths caused by pathogenic bacteria. However, these weapons of choice against multiple bacterial infections are becoming less reliable due to the lack of progress in the development and discovery of new classes of antibiotics, coupled with the emergence of antibiotic-resistant bacteria. Bacteria that are sensitive to antibiotics can acquire antibiotic resistance through changes in their DNA. A gene is a specific region of DNA that encodes for a specific protein or part of a protein. Mutations in genes are one way of changing the way proteins operate. When these proteins are target sites for antibiotics or involved in the mode of action of an antibiotic, this can cause resistance (2). Another way to develop antibiotic resistance is through what are known as mobile genetic elements. These are genes that can confer antibiotic resistance (e.g. by producing proteins that inactivate antibiotics) that are acquired from other bacteria or the environment (3).

One such bacterial pathogen that is seeing an alarming increase in antibiotic resistance is *Helicobacter pylori*. This organism is estimated to infect the stomachs of more than half of the world’s human population and is seen to be very common in developing countries, having a prevalence of up to 80% (4). In certain cases, various gastroduodenal complications may arise from this infection that puts a patient’s life at risk (5). The only way to eradicate an *H. pylori* infection is to treat with antibiotics since the bacterium is well adapted to remain propagating throughout the whole lifetime of an individual (6). However, the emergence of antibiotic resistant *H. pylori* strains is becoming a major roadblock that is causing failure in the first-line treatment regimens that are used (7).

The detection of antibiotic resistance phenotypes is important when carrying out clinical decisions for patient treatment. Unfortunately, conventional phenotypic testing for antibiotic resistance involves culturing bacteria which takes a significant amount of time. Whole-genome sequencing and genotyping methods are emerging as a fast alternative to drug resistance prediction, by considering the presence or absence of certain genes (8). Databases such as the Comprehensive Antibiotic Resistance Database (CARD) provide manually curated antibiotic-gene associations from the biomedical literature and are available online (9). In an ideal scenario, such databases would be both, exhaustive and up to date, with the latest research, which unfortunately is rarely the case. The manual process of curation makes updates to the databases slow and cumbersome especially considering the large amount of biomedical literature that is constantly being published. To help in curation, CARD employs text mining algorithms to help prioritize literature for manual reviewing (10). A text mining system that incorporates deep learning models has the potential to further improve the curation process by automatically extracting gene-antibiotic resistance relations from the biomedical literature, reducing manual reviewing.

To this end we report on the development of a methodology to automatically extract gene-antibiotic resistance relations from biomedical literature abstracts. This was then applied to extract genes related to antibiotic resistance in the bacterium *H. pylori*. The main contributions of this paper are as follows:

- We developed a novel pipeline that incorporates freely available online data in the form of English biomedical abstracts and knowledge bases, that are processed to output predicted gene-antibiotic relations that can aid in the manual curation process.
- A novel silver standard corpus dataset was developed that contains positive and negative examples of genes-antibiotic resistance relations which can be used for the development of future supervised models for gene-antibiotic resistance relation extraction.
- We compared the performance of two state-of-the-art (SOTA) models, BioBERT and PCNN, using subsets of the silver standard corpus composed of single-entity and multi-entity training dataset configurations.
- We propose potentially novel genes for metronidazole resistance in *H. pylori* using the developed pipeline. These genes were found to be linked with metronidazole resistance in other bacterial species and are candidates for further lab-based testing involving *H. pylori.*

The is organized as follows: Section 2 presents a literature review of related works; Section 3 outlines the developed pipeline in detail; Section 4 and 5 present and discuss the results respectively and lastly, Section 6 presents the conclusions of the paper.

## Related work

Relation extraction (RE) is a task of interest in natural language processing (NLP) that studies automated extraction of relations from text. Relations can be defined as triplets consisting of two entities, and an association or property that links them together (11). Entities are any words or phrases in the text that usually refer to real-world objects or concepts that are of special interest to the problem under study. Biomedical RE (BioRE) is then the extraction of relations from the biomedical domain from sources such as peer-reviewed journal publications. An example of BioRE involving two entities would be RE of protein–protein interactions, which has been extensively studied. Other than relations between entities (entity mentions), the BioRE literature also tackles relations involving either action phrases or verbs (event mentions). Here we will only focus on BioRE between two entities which can be defined as binary (e.g. an interaction that is present or absent between two proteins) or multi-class (e.g. type of interaction in drug–drug interactions).

Much of the early BioRE research was based on the extraction of protein–protein interactions (PPIs), initially using rule-based approaches that incorporated entity co-occurrences and pattern matching, as was tackled by Ng and Wong (12). Methods for automated generation of text patterns to extract relations were later studied by Huang *et al.* (13), who incorporated part-of-speech tagging to tag sentences containing PPI relations. These were subsequently used to identify new PPIs by considering sentences with similar part-of-speech tag sequences. Similarly, Thomas *et al.* (14) further improved upon this by using dependency parsing of phrases containing PPIs to automatically generate query patterns. Machine-learning-based methods for REof PPIs quickly started to be developed as an alternative to rule-based methods due to their superior performance. Miwa *et al.* (15) used a corpus weighted Support Vector Machine (SVM) to outperform other PPI-RE methods at that time. Muzaffar *et al.* (16) compared Naïve Bayes and SVM models for extracting disease-treatment relations and found that when using a mixture of lexical and sematic features, their SVM model outperformed both the Naïve Bayes model and other previously developed models tested on the same corpus.

More recently, deep learning algorithms have become the dominant approach for BioRE, which achieved SOTA performance in various tasks (11). The most common deep learning model architectures generally consist of an embedding layer to vectorize the word token inputs, a deep neural network component which is usually variants of convolutional neural networks (CNN) or recurrent neural networks (RNN), and a final fully connected layer that outputs to a Sigmoid or SoftMax function for binary or multi-class classification respectively. Word embeddings are word token representations in the form of low dimensional vectors that capture both semantic and syntactic information of words. Unsupervised models such as word2vec (17) and ELMo (18) can be pre-trained on millions of examples from PubMed and be applied to a wide variety of problems by using a dictionary that maps the word tokens to the trained vector representations. For BioRE tasks, Zeng *et al.* (19) found that concatenating word embeddings with positional embeddings increases model performance. Positional embeddings capture the relative distances of the words in a sentence compared to the entities of interest. The word and positional embeddings are then processed by the RNN/CNN-based part of the models in order to extract useful features. CNN models have been applied for various BioRE tasks including PPI (20) and drug–drug interactions (21), achieving good performance. Generally speaking, RNN methods perform better than CNN methods, but on the other hand, CNN methods are faster to train compared to deep neural networks of similar parameter size since they can be parallelized (22). Long-short term memory (LSTM) models, which are an improvement on RNN have been recently used in various BioRE tasks such as drug–drug interactions (23) and chemical–protein interactions (24). Recently, the attention mechanism has been applied to NLP in general and has helped to achieve new SOTA performance, including when used in RE. Initially, attention mechanisms were developed for encoder-decoder neural networks that were applied to language translation tasks (25). An attention mechanism helps a model to focus on important features in sequences of text, allowing it to perform better on longer stretches of text. The transformer model architecture is a recent innovation that uses multiple attention mechanisms (26). BERT, a model based on 12 transformers, achieved SOTA benchmarks in multiple RE tasks (25). BioBERT, a variant of BERT trained on biomedical and computer science literature achieved SOTA benchmarks in BioRE such as in chemical–disease interactions in the ChemProt corpus, where an impressive improvement of 7.58% in F1-score was achieved from the previous best SOTA (27).

Recent work in BioRE has been involved in the development of deep neural network architectures thanks in part to semi-supervised methods that allow large labelled datasets to be generated without the need for manual annotation (28). Progress in the development of supervised BioRE deep learning algorithms has been limited by the availability of large manually annotated corpora. Distant supervision techniques offered a solution to this problem by automatically generating large datasets with the trade-off of introducing some amount of noise. The idea of distant supervision for BioRE was first proposed by Craven & Kumlien (29) and later extended to RE in general by Mintz *et al.* (30) who framed the distant supervision assumption that if two entities are known to have a relationship, such as in KBs, sentences that contain these entities ‘might’ express this relation. The simplest application of distant supervision would be to label as true relations sentences that contain the two entities of interest and are part of a triple in a KB. This naïve approach is prone to introducing noise in the labelling process, such as in cases when the relation is not expressed in certain sentences even though these entities are present. Research later focused on reducing the noise impact of the datasets generated by distance supervision. In multi-instance learning (MIL), instances with the same entity pairs are grouped together into bags with the assumption that at least *one* instance in that bag is expressing the true relation. Zeng *et al.* (31) implemented MIL for RE by considering each instance in a bag individually where the model learns to maximize the probability of which instance in that bag most likely corresponds to the bag class label, given the current model parameters during training. The same authors also developed a novel architecture known as a piecewise CNN (PCNN) which uses piecewise max pooling instead of the conventional max pooling. This was shown to achieve better performance in RE as it better preserves important signals by separately pooling three different segments for a given vectorized sentence encoding, rather than pooling the whole vectorized sentence encoding in one go, as what happens in max pooling (31). The MIL PCNN architecture was later further improved by the inclusion of a selective attention mechanism by Lin *et al.* (32). Their model allows the inclusion of all instances in a bag rather than the single most representative instance, weighted by an attention mechanism that increases the weight of relevant instances in the bag. Beltagy *et al.* (33) used both a distantly supervised dataset and a smaller manually annotated dataset for their RE task. They noted that the models performed best when trained on both datasets simultaneously. Q. Dai *et al.* (34) took a different approach and used joint learning of two tasks—knowledge graph completion and RE, by sharing the attention mechanism between the two models, which resulted in significant improvements compared to previous approaches used in a gene-disease BioRE task. Pre-trained language model approaches, such as BERT have also been applied to MIL by aggregating the resulting instance-level vector representations by selective attention to give bag-level representations. Soares *et al.* (35) demonstrated that this type of MIL coupled with the addition of entity markers that encode for directionality of the relation, achieved better overall performance than the approach taken, Q. Dai *et al.* (34) using the same dataset.

## Methodology

### Pipeline overview

**Figure 1.**
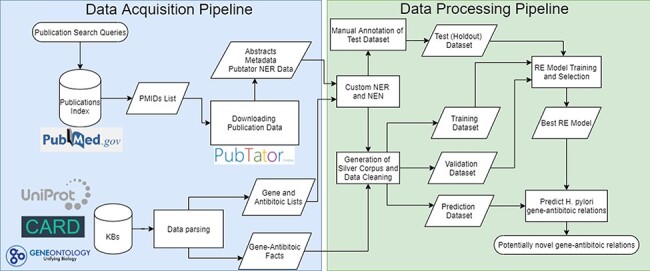
Complete flowchart of the developed pipeline which is divided as data acquisition on the left and data processing on the right.

The pipeline developed follows a typical RE pipeline with separate NLP tasks (refer to Figure 1 for a visual representation of the whole pipeline). The pipeline can be broken down as follows:

PMIDs—PubMed IDentifiers; NER—Named Entity Recognition; NEN—Named Entity Normalization.

#### Data acquisition

The documents used were in the form of English abstracts obtained from PubMed/PubTator (www.ncbi.nlm.nih.gov/research/pubtator/). The PubTator Application Programming Interface (API) was used to obtain raw abstract texts from PubMed as well as metadata related to the publications and annotations of various biomedical entities. As for the gene-antibiotic resistance facts, these were obtained from CARD (https://card.mcmaster.ca/) and UniProtKB (www.uniprot.org/).

#### Named entity recognition and named entity normalization

PubTator was used to extract gene and species mentions from the documents. A custom-built dictionary of gene identifiers and antibiotic terms was also used to increase the number of entities detected. NEN was done by linking genes to UniRef50 IDs, antibiotics to MeSH term IDs and bacterial species to NCBI taxonomic identifiers.

#### Dataset generation

A silver standard corpus was generated using distant supervision which was later used to produce training, validation and prediction datasets. An additional manually annotated testing (holdout) dataset was also produced for evaluation of the final models.

#### RE

RE was carried out using deep-learning models. Once trained, the models were evaluated on the holdout dataset.

#### Prediction of resistance genes

The best performing model was applied on abstracts containing *H. pylori* gene and antibiotic mentions. This was used to further validate the prediction outputs.

### Study datasets

#### Abstracts

English abstracts were obtained by querying PubMed (https://pubmed.ncbi.nlm.nih.gov/) using: ‘((“drug resistance bacterial”[MeSH Major Topic]) NOT (“review”[Publication Type])) AND (antibiotic OR antibacterial OR antimicrobial AND gene and resistance NOT Review[ptyp]) AND (Drug Resistance, Microbial[Mesh] AND “Bacteria/genetics”[Mesh] NOT (Review[ptyp]))’. Additionally, all literature referenced in CARD and all publications mentioning genes linked to the Gene Ontology (GO) (http://geneontology.org/) term for ‘response to antibiotic’ in UniProtKB using the query ‘goa:(“response to antibiotic [46 677]”) taxonomy: “Bacteria [2]”’ were included. The obtained list of publication identifiers (PMIDs) was then used to obtain a total of 61 219 abstracts from PubTator.

#### Knowledge bases

The CARD and the UniProt KBs were used to obtain all known gene/protein–antibiotic resistance relations. All entries in CARD are manually reviewed and are of high quality. UniProtKB contains both manual (Swiss-Prot) and automatically annotated (TrEMBL) gene entries. Moreover, UniProtKB is linked to several other biomedical databases including the GO. Linking to GO terms can occur both through manual curation and automated inferences. For the scope of this paper, all manual and automated entries were included in order to increase recall of known genes with responses to different antibiotics at the cost of including false relations. However, only those genes that explicitly stated which antibiotics were involved in their resistance were kept, which helped to keep possible false relations to a minimum. This information was obtained from the ‘Function [CC]’ field of UniProtKB which often contains references linking to publications stating those assertions.

#### Dictionaries

The NER system developed was based on dictionary searches of all known gene identifiers and antibiotics. A list of bacterial gene identifiers (gene symbols, ordered locus names and open reading frame codes) was generated from UniProtKB which resulted in 31 146 310 unique entries. The dictionary of antibiotics was generated by extracting all antibiotic terms found in CARD. Several additional antibiotic entities were manually added by the additional of plurals (e.g. carbapenem giving carbapenem**s**), and the addition of other terms of relevance such as ‘multidrug’ and ‘multi-drug’, which themselves are not antibiotics but are terms used to refer to resistance to multiple types of antibiotics. These amounted to a total of 405 antibiotic entities.

#### Generation of a silver standard corpus

NER was carried out by using the annotated output of PubTator using a custom dictionary-based approach for genes and antibiotics. Genes were normalized to their UniRef50 cluster IDs which refer to clusters where the protein sequences of each gene member in that cluster have a 50% sequence identity with the seed sequence of the cluster. By using the UniRef50 IDs, genes that are highly similar in their sequence (and consequently functionality) and that could have been referred to with different gene identifiers in the literature, can still be grouped together. For example, the gene *rdxA* found in *Helicobacter acinonychis* is also found in *H. pylori* where it is referred to as HP_0954. By using their UniRef50 ID ‘UniRef50_O25608’, both homologous genes can be linked together. Similarly, in order to reduce the granularity of antibiotics in downstream tasks, these were linked to their respective main antibiotic category as found in CARD. For instance, the antibiotic ‘carbapenem’ was normalized to ‘beta-lactams’ since it forms part of this group. All sentences with a gene and antibiotic mention were extracted using the rule-based sentence segmentation ‘sentencizer’ in the spaCy v.2.3.2 python package (https://spacy.io/). A total of 29 935 unique sentences were obtained that had at least one gene and one antibiotic mention. To increase the dataset quality, both short sentences having character lengths of less than 25 and long sentences with character lengths of more than 600 were removed. From these sentences, 81 889 candidate gene-antibiotic relations were extracted. Extracted candidate relations that were present in the KBs were labelled as ‘relation’ while those that were not present in the KBs were labelled as ‘NA’. It is reasonable to assume that not all possible gene-antibiotic resistance facts are present in the KBs used. This has the implication that multiple gene-antibiotic relations that are true but not present in the KBs will be incorrectly labelled as ‘NA’ (false negatives). To reduce incorrectly labelling these cases in the training datasets, only those candidate relations having gene entities present in the KBs were considered for labelling, while the rest were removed. Additional filtering was required to prevent several false negatives. While in the literature the term ‘multidrug’ (which is detected as an antibiotic entity in the developed NER module) can be used to refer to resistance to several antibiotics, this proved difficult to link in KBs where an explicit relation of a gene-multidrug resistance was not present. Thus, candidate relation pairs extracted from texts having genes paired to ‘multidrug’ were removed since a number of them could possibly be labelled incorrectly as ‘NA’. The only exceptions were cases where a candidate relation containing ‘multidrug’ was explicitly found in the KBs, which usually were found in UniProtKB. These were kept and labelled as ‘relation’.

#### Generation of additional datasets

Several extracted sentences contained more than one antibiotic and gene candidate relation. These sentences were expected to be more difficult for the models to learn meaningful patterns from than sentences with a single candidate relation. To test this, several different training and validation sets were produced to study the impact of the different types of data subsets used on the models’ performances. Table 1 describes the different datasets generated to train the models.

**Table 1.** Description of the different dataset subsets used for model training

Dataset code	Dataset description	Training counts (% positive labels)	Validation counts (% positive labels)
SINGLE	Candidate relations from sentences containing only a single candidate relation	8828 (89 %)	1528 (88 %)
MULTI	Candidate relations from sentences containing only multiple candidate relations	37 002 (69 %)	8351 (54 %)
MULTI_LIMITED	Candidate relations from sentences containing multiple relations with the genes belonging to a maximum of 2 UniRef50 cluster IDs and the antibiotics belonging to a maximum of 2 different antibiotic groups	28 961 (87 %)	4712 (81 %)
FULL	All candidate relations from all sentences	45 830 (73 %)	9879 (60 %)

The train/validation split was achieved by keeping all sentences extracted from abstracts published in the period 2018–20 as part of the validation sets, and the rest as the training sets. An additional testing (holdout) dataset was manually annotated by an expert in antibiotic resistance using the prodigy (https://prodi.gy/) annotation toolkit, using 600 randomly selected candidate relations obtained from unique sentences not present in the training and validation datasets, of which 541 were used following further manual inspection. When grouped by gene-antibiotic entity pairs (using the gene UniRef50 ID and the antibiotic main group ID) the testing dataset consisted of 186 bags with the ‘relation’ label and 235 bags with the ‘NA’ label (see Table 2). To validate the predicted outputs, a prediction dataset was generated by obtaining a list of *H. pylori* genes from UniProtKB which were then used to extract all sentences having genes with the same UniRef50 IDs as the *H. pylori* genes. To discover new relations, only candidate relations that were not present in the KBs were kept in the final prediction dataset. This amounted to a total of 4976 candidate relations.

**Table 2.** Summary of holdout dataset

Dataset grouping	Relation	NA
Single-instance	235 (43 %)	306 (57 %)
Multi-instance (bags)	186 (48 %)	203 (52 %)

### RE methods

The implementations of the deep learning models in the OpenNRE v0.1 (https://github.com/thunlp/OpenNRE). python package were used for this task. A total of 4 different model configurations were used (see Table 3). Refer to Supplementary Appendix A for additional details of the model parameters used.

**Table 3.** Summary of deep learning models used

Model code	Model type
PCNN	Piecewise Convolutional Neural Network (PCNN) with pretrained word2vec word vectors
BIOBERT	BioBERT uncased base model with pretrained weights with entity markers
BAG_PCNN	Multi-instance learning PCNN using pretrained word2vec word vectors and with instances having the same entity pairs grouped in bags.
BAG_BIOBERT	Multi-instance learning BioBERT uncased base model with pretrained weights and with instances having the same entity pairs grouped in bags.

The PCNN models are based on the implementations of Zeng *et al.* (31) with piecewise max pooling that showed increased performance for RE tasks compared to conventional max-pooling, while the pretrained BioBERT (https://github.com/dmis-lab/biobert) model was based on the implementation of Soares *et al.* (35) that uses additional entity markers to help the model increase attention on the entities of interest. Both multi-instance and single-instance models were tested to compare their performance on the different datasets since multi-instance models are known to generally perform better in noisy datasets. The output layer of all models was a fully connected layer that outputs a single value using a sigmoid function to normalize the result between 0 and 1. This score was then used to label examples based on the threshold of 0.5 where examples are labelled relation if the score was ≥ 0.5 and NA if the score was < 0.5.

The PCNN neural networks required word vectors as inputs from the texts. To this end, the word2vec pretrained embeddings of Zhang *et al.* (22) were used. Since these word embeddings are very large and did not fit in memory with the hardware available, the pretrained embeddings were reduced to include only the top 1 million most frequent tokens. The use of both the pretrained word2vec word embeddings and the pretrained BioBERT model weights allowed the introduction of additional rich information that would have otherwise not been included by simply using the limited datasets obtained from the generated silver standard corpus.

### Evaluation

Evaluation of the trained models was done on the holdout dataset at the bag-level, to directly compare both the multi-instance and single-instance level models. Since the single-instance models do not output a label for a bag, this was indirectly obtained by first obtaining predictions of all the instances in a bag and then using the top-scoring prediction in that bag to specify the bag label, which was the same approach used by Ray and Craven (36). The evaluation metrics used were recall, precision and F1-score. For selecting the best model, the F1-score was favoured as it incorporates both precision and recall into a single metric and is robust to label class imbalance.

**Figure 2.**
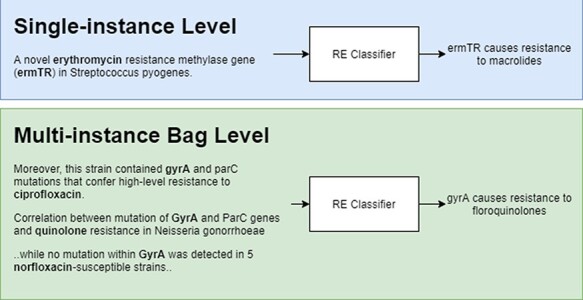
Single-instance vs multi-instance RE.

Figure 2 shows a representation of model predictions in single-instance vs multi-instance learning with the former giving a prediction for each instance and the latter grouping instances (in bags) with the same entity pairs (genes and antibiotics) and giving a single prediction for each bag. During training, bags are labelled as ‘relation’ if there is at least one positive instance, and ‘NA’ if all instances are labelled as *NA*.

## Results

### Silver standard corpus

**Figure 3.**
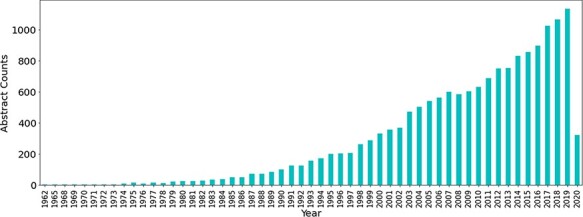
Number of publications related to antibiotic resistance till Q1 of 2020.

Initially a total of 60 490 abstracts related to antimicrobial resistance were obtained from PubTator using the PubMed IDs obtained from the PubMed queries, the majority of which have been published in the last 2 decades. The number of publications related to antibiotic resistance has seen a large increase throughout the years (see Figure 3).

From the 60 490 abstracts obtained, 22 915 (38%) contained sentences with both a gene and antibiotic mention. Sentences containing both entities are sentences that potentially express a relation between the genes and antibiotics mentioned. This study only considered sentence-level relations, at the cost of missing any gene-antibiotic relations which are expressed between multiple sentences. In fact, an additional 20 349 (34%) abstracts contained gene and antibiotic mentions without ever occurring in the same sentences. Table 4 below gives a summary of the abstracts obtained with associated statistics.

**Table 4.** Summary of the abstracts obtained from PubMed related to antibiotic resistance

Description	Statistic
No. of abstracts	60 490
No. of abstracts with sentences having both gene and antibiotic mentions	22 915 (38 %)
Mean no. of unique genes per abstract	3.6 (± 2.9 SD)
Mean no. of unique antibiotics per abstract	3.5 (± 2.9 SD)
Mean no. of characters in the abstracts’ text	1485.8 (± 596.1 SD)
Mean no. of tokens in the abstracts’ text	209 (± 85.4 SD)

After segmenting the abstracts into different sentences, a total of 29 935 unique sentences were obtained that contained both gene and antibiotic mentions. On average, these sentences had more than one unique gene and antibiotic mention (see Table 5).

**Table 5.** Summary of the sentences having both gene and antibiotic mentions

Description	Statistic
No. of unique sentences	29 935
Mean no. of tokens in sentences	26.9 (± 11.4 SD)
Mean no. of unique gene mentions per sentence	1.5 (± 1.1 SD)
Mean no. of unique antibiotic mentions per sentence	1.4 (± 1.0 SD)

**Figure 4.**
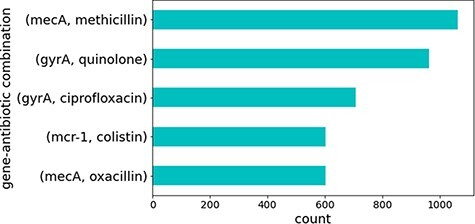
Top five gene identifiers and antibiotic combinations found in sentences.

Several sentences contained combinations of the same genes and antibiotics. These represented well-studied genes that are known to cause antibiotic resistance. Figure 4 illustrates the top five gene identifiers and antibiotic combinations found in these sentences, all of which are known to be true relations of genes that cause resistance to the specified antibiotics in CARD. This is no surprise since genes and antibiotics which co-occur multiple times in different sentences and publications would be expected to have some sort of relation.

All possible combinations of gene and antibiotic mentions found in the different sentences were all candidate relations. Each sentence could have multiple candidate relations depending on the number of possible combinations of gene and antibiotic entities present. The example below was obtained from PubTator and illustrates a sentence having two candidate relations which in this case were positive relations.

**Table:** 

**PMID: 32335280** **Sentence:** OBJECTIVES: This study aimed at identifying and characterizing *oxazolidinone* resistance genes *cfr* and *optrA* in Enterococcus isolates. **Entities**: (crf—Gene), (optrA—Gene), (oxazolidinone—Antibiotic) **Candidate relations:** <crf, oxazolidinone>, < optrA, oxazolidinone >

A total of 81 889 candidate relations were obtained from all sentences, out of which 4976 were identified as being related to *H. pylori*. Table 6 contains summary statistics for the extracted candidate relations.

**Table 6.** Summary of candidate relations obtained from sentences containing both gene and antibiotic entities

Metric	Score
Precision	0.87
Recall	0.63
F1-score	0.73

The quality of the silver standard corpus was evaluated using the manually annotated holdout dataset (refer to Table 7). A degree of false-positives was expected to be found since this dataset was automatically generated, but the cost of introducing false-positives was balanced by the huge benefit of achieving a larger dataset that would not have been possible by manual annotation, since the process is very time-consuming and labour-intensive. The silver standard corpus contained only already known relations that are found in KBs. The supervised models developed improved upon this by learning patterns from examples in the silver standard corpus that were applied to detect novel relations that are currently not present in the KBs used to generate the silver standard corpus.

**Table 7.** Evaluation of the rule-base method used for generating the silver standard corpus using the holdout dataset

Description	Statistic
No. of candidate relations	81 889
No. of unique candidate relations	11 625
No. of candidate relations related to *H. pylori* genes	4976
No. of unique candidate relations related to *H. pylori* genes	1434

### KB

Known gene-antibiotic relations (facts) were obtained from the CARD and UniProt KBs. These amounted to 42 380 unique facts when considering all gene identifiers and antibiotic names, and 2455 unique facts when considering Gene UniRef50 IDs and antibiotic groups (refer to Table 8).

**Table 8.** Summary of knowledge bases composition of genes, antibiotics and associated facts

Description	Statistic
No. of unique facts (gene identifier and antibiotic name)	42 380
No. of unique facts (gene UniRef50 ID and antibiotic group)	2455
Gene identifiers	
‘No. of unique gene identifiers’	35 905
‘Mean no. of associated antibiotics with each gene identifier’	1.2 (± 0.5 SD)
‘Mean no. of associated antibiotic groups with each gene identifier’	1.1 (± 0.4 SD)
UniRef50 Clusters	
‘No. of unique UniRef50 Clusters’	2147
‘Mean no. of associated antibiotics with each Uniref50 Clusters’	1.6 (± 1.2 SD)
‘Mean no. of associated antibiotic groups with each Uniref50’ ‘Clusters’	1.1 (± 0.6 SD)
Antibiotics	
‘No. of unique antibiotics’	121
‘No. of unique Antibiotic groups’	35

### Model evaluations

Table 9 shows the evaluation metrics for the different deep-learning models trained on the different training datasets. The single-instance models (BIOBERT and PCNN) were evaluated on bag-level predictions to be directly comparable to the multi-instance models. Overall, the BIOBERT models achieved the highest F1-scores for all datasets except for the model trained on the SINGLE dataset, where the BAG_BIOBERT model achieved a slightly higher F1-score than the other models trained on the same dataset. The rest of the models trained on the SINGLE dataset failed to achieve a meaningful decision boundary and simply classified all examples as ‘relation’, achieving the equivalent F1-score of a co-occurrence model. BERT-based models are well known to achieve SOTA performance in various tasks and are also seen to perform generally better than PCNN based models for this task. The multi-instance models achieved slightly higher precision scores than the single-instance models, which in turn achieved higher recall scores. Moreover, the single-instance models performed better than the multi-instance models in all cases (when excluding the models trained on the SINGLE dataset). The best performing multi-instance model was the BAG_BIOBERT model trained on the SINGLE dataset with an F1-score of 0.68. The BIOBERT model trained on the MULTI dataset achieved the highest F1-score of 0.74 which was 0.01 points higher than that of the same model trained on the FULL dataset and was the best performing model overall having achieved 0.09 points higher than the baseline co-occurrence model.

**Table 9.** Holdout dataset metrics of all models tested on different datasets

Dataset and model used for training	Precision	Recall	F1-score
Co-occurrence (bag level)	0.48	1.00	0.65
FULL			
*BAG_BIOBERT*	0.62	0.55	0.58
*BAG_PCNN*	0.65	0.56	0.60
*BIOBERT*	0.59	0.95	0.73
*PCNN*	0.55	0.98	0.70
MULTI			
*BAG_BIOBERT*	0.61	0.62	0.62
*BAG_PCNN*	0.62	0.63	0.63
*BIOBERT*	0.60	0.95	0.74
*PCNN*	0.56	0.97	0.71
MULTI_LIMITED			
*BAG_BIOBERT*	0.65	0.66	0.66
*BAG_PCNN*	0.61	0.66	0.63
*BIOBERT*	0.56	0.94	0.70
*PCNN*	0.52	0.99	0.68
SINGLE			
*BAG_BIOBERT*	0.55	0.88	0.68
*BAG_PCNN*	0.48	1.00	0.65
*BIOBERT*	0.48	1.00	0.65
*PCNN*	0.48	1.00	0.65

### Prediction of *H. pylori* genes associated with antibiotic resistance

To further validate the pipeline, we applied it to extract gene-antibiotic relations for *H. pylori*. From the 4976 candidate relations, 3369 were predicted to contain true relations (67.7%). All these candidate relations were not present in the KBs used at the time of writing. The number of unique gene-antibiotic relations amounted to 956 out of which 750 (78.5%) were not found in sentences obtained from abstracts of publications studying *H. pylori*, suggesting that these relations have not been directly studied in association with *H. pylori.* However, this was still limited by mentions in the abstracts and does not exclude the possibility of these relations being mentioned in the main body of other publications studying *H. pylori.*

**Figure 5.**
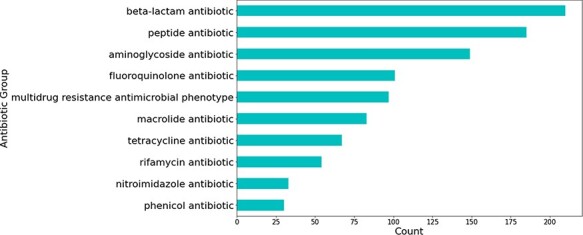
Number of predicted gene-antibiotic resistance associations linked to *H. pylori* for the top 10 antibiotic groups.

**Figure 6.**
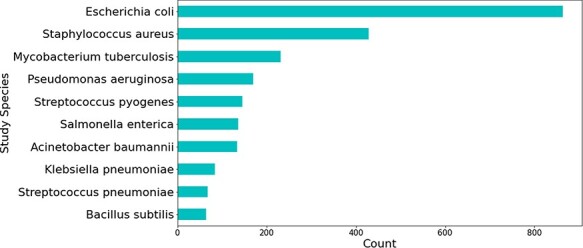
Top 10 bacterial species which were the main organism understudy in publications mentioning genes linked to *H. pylori.*

Most relations obtained were associated with beta-lactam antibiotics, followed by peptide and aminoglycoside antibiotics (see Figure 5). The relations that were not extracted from studies involving *H. pylori* were mostly obtained from those studying *Escherichia coli—*a highly researched organism that is used for a multitude of different experiments, including antibiotic resistance experimentation (see Figure 6).

**Figure 7.**
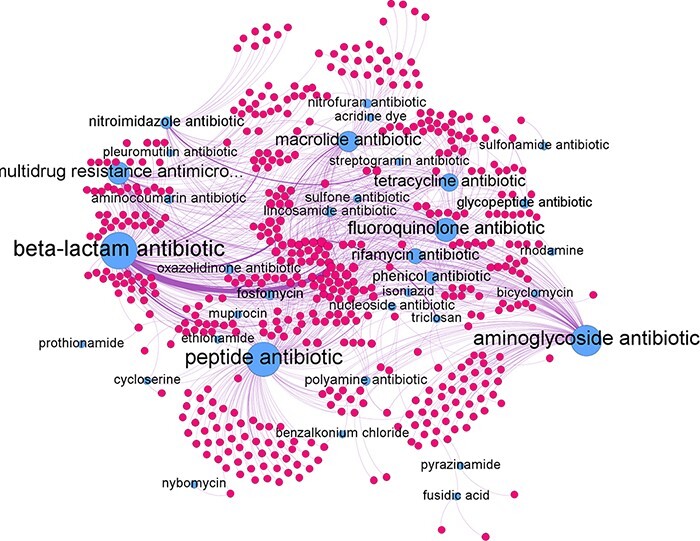
Network graph of all predicted gene-antibiotic group relations for antibiotic resistance in *H. pylori*, with blue nodes represent the antibiotic groups and red nodes represent the individual genes.

The predicted relations can be represented as a network graph (see Figure 7). In such a graph the nodes represent genes and antibiotic groups. An edge between the two represents a predicted antibiotic resistance relation. The antibiotic groups had several different edges connecting them to individual genes. In turn, each gene could be connected to several different antibiotics groups if it confers resistance to multiple antibiotic groups. The average degree of edges connected to the antibiotic group nodes was found to be 24.2 with the highest degree of 151 achieve by the beta-lactam antibiotic node and the lowest of 1 by the pleuromutilin and the prothionamide antibiotic groups. For individual antibiotic group subgraphs refer to Supplementary Appendix B.

### Negations

Associations between genes and antibiotic resistance occurred either as absence of genes that cause resistance (association via absence) or presence of genes that cause resistance (association via presence). In either case, an association is present. From manual reviewing of sentences, whenever negation occurred, it was used to indicate cases of association via absence or complete lack of an association. Using the python package negspaCy v.1.0.0 we obtained estimates for the prevalence of negations in the training dataset as being 7.6% and 5.0% for positive (relation) and negative (NA) samples respectively and in the case of the prediction dataset as being 9.7% and 8.2% for positive (relation) and negative (NA) samples respectively. Thus, the negation prevalence estimates in both the training and predictions were low. In the case of the predictions, certain negated sentences were incorrectly predicted as having an association such as:

**Table:** 

**PMID:** 16953176 **Prediction**: relation **Text:** ‘… we discovered no relationships between iceA genotypes and functional dyspepsia or duodenal ulcer, nor between *clarithromycin* resistance and *iceA* genotypes.’

However, other negation sentences were also correctly predicted as having no relation:

**Table:** 

**PMID:** 28328947 **Prediction**: no relation (NA) **Text:** ‘Although it was not resistant to *ciprofloxacin*, the *kdsD* mutant shared many phenotypic characteristics with the CipR mutant…’

Moreover, cases of negation expressing association via absence were correctly predicted:

**Table:** 

**PMID:** 28533243 **Prediction**: relation **Text:** ‘Synergistic effects were observed in strains harboring no *ramR* gene and a mutated tet(A), with an 8-fold increase in the *tigecycline* MIC…’

## Analysis of results

While RE has been studied for various biomedical tasks it has not been investigated in the field of antibiotic resistance research. Instead, several studies take a different approach by utilizing gene/protein sequence data obtained from genomic sequencing, with more recent approaches employing machine learning and deep learning to predict similar genes that have the same antibiotic resistance phenotype (37). RE of gene-antibiotic resistance relations has unique challenges. First and foremost, there is a lack of gold standard corpora of annotated datasets that can be used to train and evaluated high-quality models. In this study this problem was tackled using distant supervision which produced an automatically labelled silver standard corpus dataset. Fortunately, there are a few KBs that contain gene-antibiotic relations that can be used for such purpose. However, distant supervision introduces noise in the labelling process. Several different RE approaches and models have been proposed throughout the last decade to tackle the noise problem in distant supervision, such as multi-instance learning (MIL) that uses bags as model inputs instead of single instances. From the results obtained, the MIL models used in this study performed poorly during evaluation (see Table 9). In fact, the multi-instance models (BAG_BIOBERT and BAG_PCNN) obtained lower holdout dataset F1-scores on all training datasets, compared to the single-instance models (when excluding the SINGLE dataset). One possible reason why these models performed worse could be due to the relatively small dataset sizes used. A small dataset is challenging for a complex model to learn from, such as the relatively more complex multi-instance models, especially when considering that SOTA performance of these models was observed in datasets with hundreds of thousands to millions of examples (e.g. see 34).

To further reduce the impact of noise, several different subsets of the original full dataset were used to train different models to see if any subset would perform better. This was driven by the assumption that certain examples in the dataset, such as sentences containing multiple gene and antibiotic entities, were more likely to be mislabelled via distant supervision than sentences with a single mention of a gene and an antibiotic entity. Sentences with multiple different antibiotic and gene entities were highly prevalent (see Table 1 MULTI vs SINGLE dataset sizes) which made the task of RE significantly harder. For instance, in the example below, there are a total of 14 candidate pairs out of which 10 were true relations and four are false relations. The best model used (BIOBERT trained on the MULTI dataset) classified all as ‘relations’ in this case, erroneously labelling an additional four relations as true.

**Table:** 

**PMID:** 15722450 **Text:** The G262S point mutation distinguishing the metallo-beta-lactamase *IMP-1* from *IMP-6* has no effect on the hydrolysis of the drugs *cephalothin* and *cefotaxime*, but significantly improves catalytic efficiency toward *cephaloridine, ceftazidime, benzylpenicillin, ampicillin*, and *imipenem*. **True relations:** <IMP-1, cephaloridine >, <IMP-1, ceftazidime >, <IMP-1, benzylpenicillin >, <IMP-1, ampicillin >, <IMP-1, imipenem >, <IMP-6, cephaloridine >, <IMP-6, ceftazidime >, <IMP-6, benzylpenicillin >, <IMP-6, ampicillin >, < IMP-6, imipenem > **False relations:** <IMP-1, cephalothin>, <IMP-1 cefotaxime >, <IMP-6, cephalothin>, <IMP-6 cefotaxime >

The dataset sizes were also suspected to be limiting the models’ performance. For instance, the BIOBERT model trained on the SINGLE dataset failed to produce any meaningful decision boundary and simply classified every instance as a true relation (like the co-occurrence model). The largest dataset used for training, which was the FULL dataset, contained 55 709 instances. In the field of deep learning this is still considered as a small dataset size. In fact, in other BioRE tasks such as protein–protein interactions Hong *et al.* (38) obtained 480 K instances through distant supervision. Unfortunately, this is simply a function of the number of publications published on the topic tackled for the RE task, which in this case is much higher for protein–protein interactions than antibiotic gene resistance. The effect of the small dataset sizes was attempted to be mitigated using pre-trained models and word embeddings. The fact that nearly all models trained on the FULL dataset performed slightly worse than those trained on the MULTI dataset shows that the instances in the SINGLE dataset were being detrimental to model performance, since the FULL dataset was simply a combination of both datasets. It is possible that there was a higher proportion of noisy labels in the SINGLE dataset compared to the MULTI dataset.

### *H. pylori* genes related to antibiotic resistance—the case of metronidazole

To assess the output of the best performing model and its usefulness in uncovering potentially novel genes associated with antibiotic resistance, we choose the bacterium *H. pylori* for testing the model’s output. All *H. pylori* genes (including genes from other species that are similar in sequence) that were predicted by the model to be associated with resistance to the antibiotic group nitroimidazole were manually reviewed. This group was chosen because one of its members, metronidazole, is a very important antibiotic for the treatment of *H. pylori*. Metronidazole resistance can arise from various mechanisms and while various resistance-causing genes have been investigated, certain studies ascertain the possibility of other unknown genes contributing to resistance (39).

**Figure 8.**
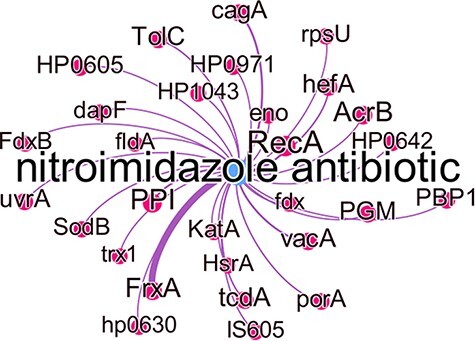
Subgraph of the nitroimidazole antibiotic group with predicted gene relations where the edge thickness is proportional to the number of times this association was predicted.

Figure 8 shows all predicted genes which were associated with nitroimidazole resistance, with the edge thickness being proportional to the number of times this association was predicted. While some of these genes are well known to cause antibiotic resistance in the literature, they were not represented in the KBs used at the time of writing. The gene *frxA* has the highest number of predicted relations with this antibiotic group and in fact is well known to be associated with metronidazole resistance in the literature (40). Other well-known genes associated with metronidazole resistance are *cagA, recA, hefA* and *vacA* all of which have experimental evidence to indicate this (40). *PPI* which in most studies refers to ‘proton pump inhibitor’ and *PGM* which was used as an abbreviation for metronidazole in one study, were incorrectly identified as gene entities.

The *katA* gene was extracted from a sentence expressing a negation in the relation and was also wrongly predicted to be associated with metronidazole resistance:

**Table:** 

**PMID**: 10543743 DNA transformation, PCR-based restriction analysis, and DNA sequencing collectively showed that the *metronidazole* resistance of this strain was due to mutation in rdxA (gene HP0954 in the full genome sequence of *H. pylori* 26695) and that resistance did not depend on mutation in any of the other genes that had previously been suggested: catalase (*katA*), ferredoxin (fdx), flavodoxin (fldA), pyruvate:flavodoxin oxidoreductase (porgammadeltaalphabeta), RecA (recA), or superoxide dismutase (sodB).

Similarly, the *def* gene was also wrongly predicted to be associated with metronidazole resistance:

**Table:** 

**PMID**: 21575176 Large divergence was seen in genes related to antibiotics: frxA (*metronidazole* resistance), *def* (peptide deformylase, drug target), and ftsA (actin-like, drug target).

The rest of the genes all had a single predicted association. Manual reviewing of the literature showed that these genes are mentioned in the main text of publications evaluating *H. pylori* metronidazole-resistance but were not detected in their abstracts (4, 40–47), apart from *uvrA, tcdA, porA*. These three remaining genes were found in sentences expressing an association with metronidazole in studies concerning other bacteria:

**uvrA**

**Table:** 

**PMID**: 2194230 **Species under study**: *Bacteroides fragilis* Genes from *B. fragilis* Bf-2 were cloned on a recombinant plasmid pMT100 which made E. coli AB1157 and *uvrA*, B, and C mutant strains more resistant to *metronidazole*, but more sensitive to far uv irradiation under aerobic conditions.

**tcdA**

**Table:** 

**PMID:** 26048022 **Species under study**: *Clostridium difficile* All of the **metronidazole**-resistant strains belonged to *tcdA* +/tcdB + genotype with triple or quintuple drug resistance phenotypes.

**PorA**

**Table:** 

**PMID:** 15150173 **Species under study:** *Bacteroides fragilis* Mutant strains lacking the genes for flavodoxin and *PorA* were less susceptible to *metronidazole* than the sensitive parent, and a double flavodoxin/PorA mutant had even less susceptibility but none of the mutants were as resistant as the spontaneous metronidazole-resistant strain.

Since these genes have not yet been described to be associated with *H. pylori* antibiotic resistance in the literature but are known to participate in resistance in other species, they are potential candidates for further lab-based studies involving *H. pylori* to verify whether they also cause similar resistance in *H. pylori*. Associations do not mean casual relationships, especially in cases of single studies, but they are drivers of further investigations. Additional predicted relations can be viewed in Supplementary Appendix B for the other antibiotic groups. This case study shows that our pipeline works well in a real-world scenario by uncovering potentially overlooked antibiotic resistance genes that have been published in the literature. While the high false-positive rate does incur additional work to manually review and filter out, our pipeline was still able to label several *NA* relations correctly, which is still a significant improvement over the alternative of using a simple co-occurrence model or manual curation, while also maintaining a relatively high recall rate.

### Limitations of the study and future work

Future work needs to tackle the shortcomings mentioned in the previous sections, namely the development of novel manually annotated gold standard corpora for validating models and increased dataset sizes used for training. Moreover, there is a need for developing models better suited for this domain rather than simply utilizing models that have shown success in other domains. From the results obtained it appears that in certain cases there was not enough information conveyed in the sentences to differentiate between true and false relations of this type. Since it appears that from the sources used in this project, the number of training examples is limited to less than 60 K, additional information outside the text at the sentence level is necessary to further improve models’ performance. One such solution would be to include additional information in the form of KB embeddings of the gene and antibiotic entities. KB embeddings have recently been studied and used within RE frameworks (34). The Gene Ontology repository is a freely accessible KB that contains annotations for gene functions. By using KB embedding algorithms, the rich information captured by the KB embedding for a particular gene and antibiotic could help evaluate whether an antibiotic resistance relation between the two is possible by considering the gene’s function and the antibiotic’s mode of action. Additionally, gene/protein sequence data could also be incorporated. The genes and proteins themselves are composed of nucleotide and amino acid sequences respectively. Many of these sequences are freely accessible through many online repositories such as NCBI and UniProtKB. In this project, we utilized the UniRef50 cluster IDs in order to indirectly capture additional associations by sharing the associations of individual gene members with all the members of the cluster. It is well known that genes that have the same functionality (e.g. antibiotic resistance) across different species can be homologs of each other and thus have a degree of sequence similarity. The UniRef50 cluster IDs clusters genes with a sequence similarity threshold of 50% or greater. Studies have shown that lower thresholds may be required in the case of genes conferring antibiotic resistance (37). Moreover, models that automatically adjust the sequence similarity thresholds to be used depending on the type of gene/protein sequences have been already developed and shown promising results (37). Research in merging sequence models with RE models could potentially create pipelines with better predictions.

Since the datasets obtained were of relatively small size, using just English biomedical abstracts as a source, the next step would be to also utilize full publications from the Medline PMC repository which should significantly increase the size of the datasets obtained through distant supervision. Moreover, a larger manually annotated dataset would also help in developing better-performing models. Studies have shown that training a model on a combined distantly annotated and manually annotated dataset can increase performance (33). These larger datasets would also benefit from an additional exploratory analysis of the distribution of sentence structures and similarities, that would provide further insights to help filter sentences with true relations and improve the quality of the silver standard corpus. Furthermore, while the final holdout set was manually annotated, this study relied on a distantly annotated validation set to monitor model performance during the actual training. Using a manually annotated validation set would have allowed for more reliably tuned and optimized models since a manually annotated validation set would have resembled real-world examples much more than the noisy distantly annotated one. Finally, the lack of past research that could be used for comparison and evaluation of our method is a limiting factor of this study. Through this work and the generation of the silver standard corpus, we hope to inspire further studies on this particular REtask and the creation of online tools similar to that Ren *et al.* (48) that would be beneficial for future research and curation efforts.

## Conclusion

To the best of our knowledge, this paper presented the first attempt of applying BioRE for genes that are associated with antibiotic resistance using biomedical abstracts. We developed a new NLP pipeline for biomedical RE of gene-antibiotic resistance relations, that included data acquisition via PubMed and PubTator, NER using PubTator complimented with custom dictionaries and RE through distant supervision using current SOTA models. Since no pre-annotated corpora of gene-antibiotic relations were available, a silver standard corpus was developed by using the facts present in the CARD and UniProt KBs. Multiple models were then trained using different subsets of this dataset. Multi-instance learning models that are known to perform well in other domains when using distant supervision were shown to perform underwhelmingly for the task of gene-antibiotic resistance RE. Shortcomings and future improvements to model performance were discussed. Nevertheless, the BioBERT model, trained on the MULTI dataset subset of the silver standard corpus, was found to perform well having an F1-score of 0.74, being 0.09 points higher than the baseline co-occurrence model. To further test the generalisability of this pipeline and the model’s output, the best performing model was used to predict whether candidate gene-antibiotic resistance relations of *H. pylori* which were not previously present in the KBs, were true or false relations. Using the nitroimidazole antibiotic group as a case study, the predicted genes related to this antibiotic group were manually reviewed. Out of a total of 28 unique genes, 2 were NER errors, 23 were found in studies concerning *H. pylori* that should be included in KBs and 3 genes were found to be studied for metronidazole resistance in other organisms only, making them good candidates for further lab-based experimental studies. From this case study, the pipeline showed great potential for aiding in the curation process for KBs/databases such as CARD. Further studies in this direction are perceived to have the potential to improve upon the results of this paper.

## Supplementary Material

baab077_Supp
