# Supplementary material for: Automated extraction of genes associated with antibiotic resistance from the biomedical literature
Source: Database (Oxford). 2022 Jan 20;2022:baab077. doi: 10.1093/database/baab077 (PMC9263533; doi:10.1093/database/baab077)

Appendix A

The details of the hyperparameters used during training for every model can be viewed in Table 10 below.

**<Table 10** – Hyperparameters of models used>

| Model Code | Model Description and Associated Python Classes | Hyperparameters |
| --- | --- | --- |
| PCNN | Instance level Piecewise Convolution Neural Network (OpenNRE v.0.1 implementation)  opennre.encoder.**PCNNEncoder**  opennre.model**.SoftmaxNN**  opennre.framework**.SentenceRE** | **PCNNEncoder:** *max_length=70, word_size=200, position_size=5,hidden_size=50, blank_padding=True, kernel_size=3, padding_size=1, dropout= 0.5, mask_entity=True* **SentenceRE:** batch_size=254, max_epoch=50, lr=0.0001, weight_decay=1e-5, opt='sgd' |
| BIOBERT | Instance level pre-trained (on biomedical literature) Bert Model  opennre.encoder.**BERTEntityEncoder**  opennre.model**.SoftmaxNN**  opennre.framework**.SentenceRE** | **BERTEntityEncoder:** max_length=70, mask_entity=True **SentenceRE**: batch_size=72, max_epoch=7, lr=2e-7, opt="adamw" |
| BAG_PCNN | Bag level Piecewise Convolution Neural Network  opennre.encoder.**PCNNEncoder**  opennre.model**.SoftmaxNN**  opennre.framework.**BagRE** | **PCNNEncoder:** *max_length=70, word_size=200, position_size=5,hidden_size=50, blank_padding=True, kernel_size=3, padding_size=1, dropout= 0.5, mask_entity=True* **BagRE:** *batch_size=128, max_epoch=50, lr=0.001, weight_decay=1e-5, opt='sgd', bag_size=12* |
| BAG_BIOBERT | Bag level pre-trained (on biomedical literature) Bert Model  opennre.encoder.**BERTEntityEncoder**  opennre.model**.SoftmaxNN**  opennre.framework.**BagRE** | **BERTEntityEncoder:** *max_length=70, mask_entity=True*  **BagRE**: *batch_size=12, max_epoch=7, lr=0.0000002, weight_decay=1e-5, opt="adamw", bag_size=12* |

Appendix B

Figures 9-15 are the predicted relations of *H. pylori* genes causing antibiotic resistance. These predicted relations were not present in CARD or UniProtKB at the time of writing.

<Figure 9 - Predicted genes related to fluoroquinolone antibiotic resistance in *H. pylori* (74 unique genes).>


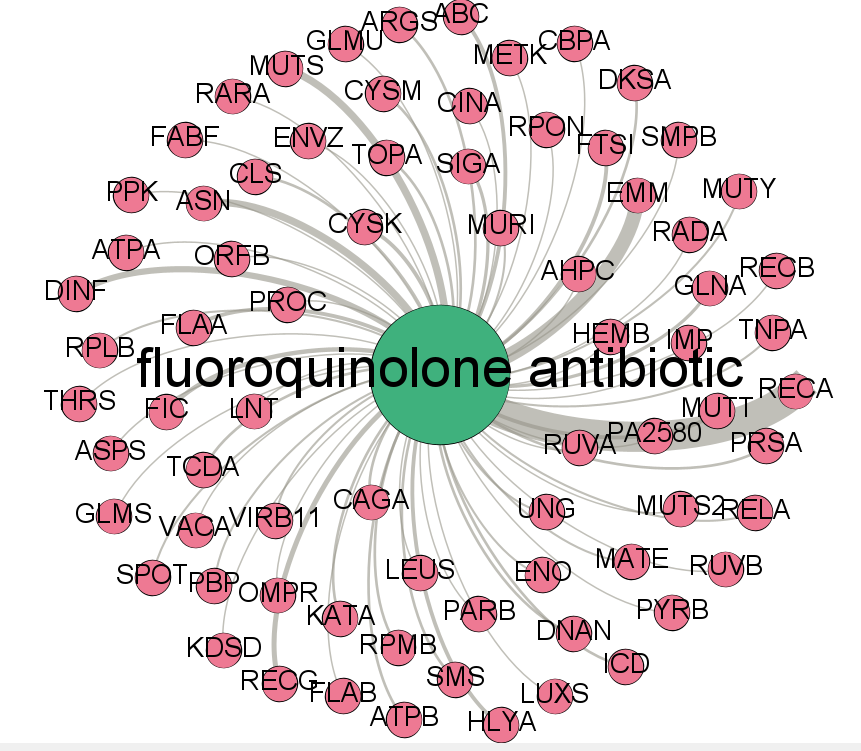


**<Figure 10** - Predicted genes related to macrolide resistance in *H. pylori* (55 unique genes).>


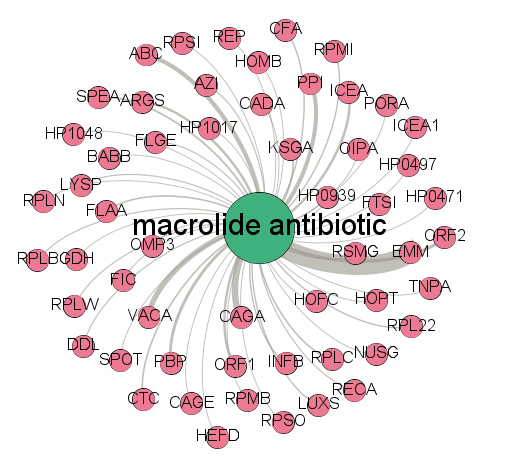


<Figure 11 - Predicted genes related to beta-lactams resistance in *H. pylori* (145 unique genes).>


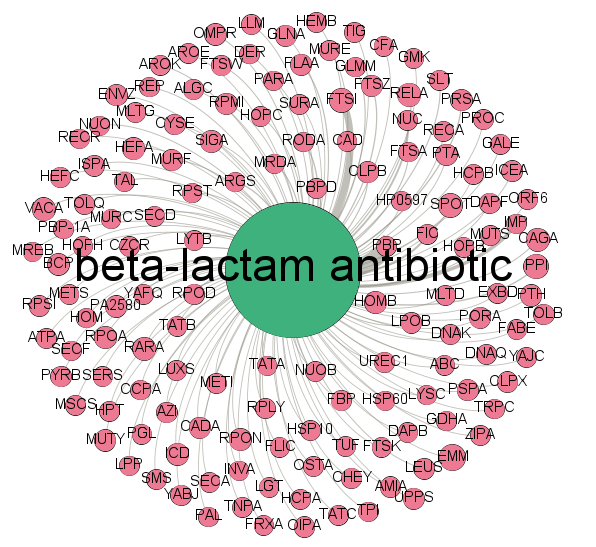


<Figure 12 – Predicted genes related to aminoglycoside resistance in *H. pylori* (94 unique genes).>


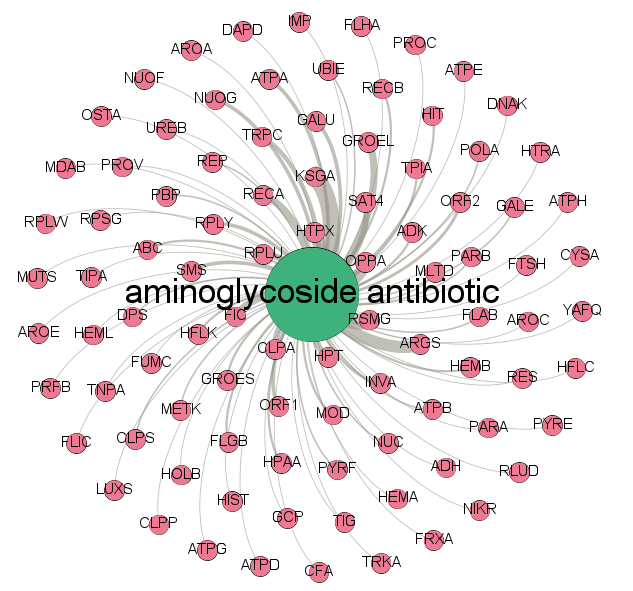


<Figure 13 - Predicted genes related to multidrug resistance in *H. pylori* (72 unique genes).>


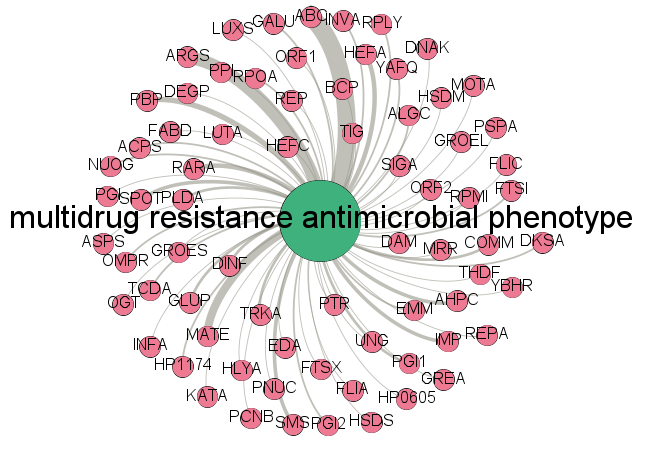


<Figure 14 - Predicted genes related to rifamycin resistance in *H. pylori* (37 unique genes).>


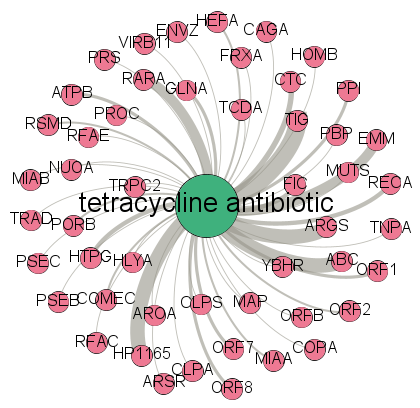


<Figure 15 - Predicted genes related to tetracycline resistance in *H. pylori* (50 unique genes).>


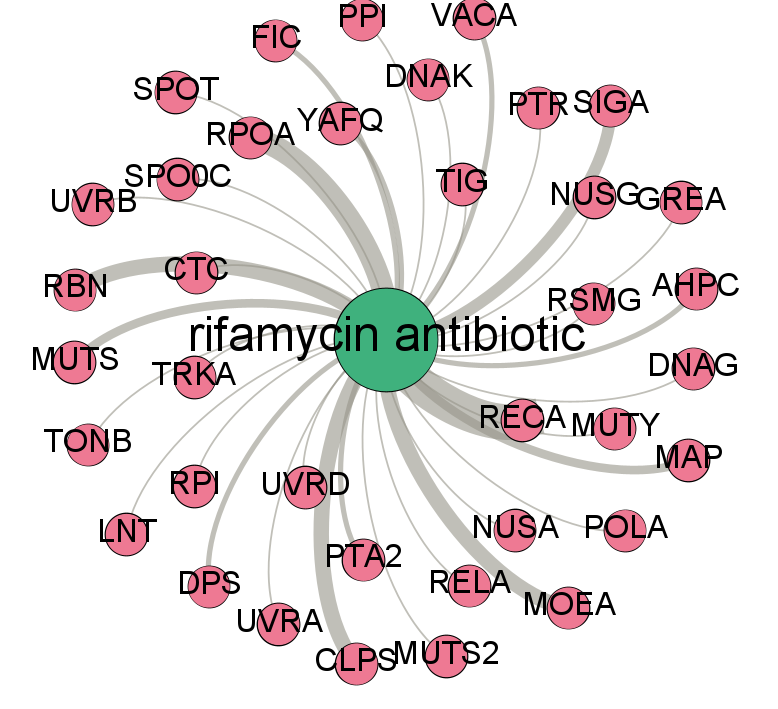

Supplement: baab077_Supp [file baab077_supp.zip › Supplementary_rev_final_no_links.docx]
